# Guild Structure in Coral Reef Fishes Does Not Conform to Competitive Exclusion–Based Assembly Rules

**DOI:** 10.1002/ece3.74011

**Published:** 2026-07-09

**Authors:** Simon P. Um, John W. Turnbull, Emma L. Johnston, Graeme F. Clark

**Affiliations:** ^1^ School of Life and Environmental Sciences, Faculty of Science University of Sydney Sydney New South Wales Australia; ^2^ Securing Antarctica's Environmental Future (SAEF) Sydney New South Wales Australia

**Keywords:** assembly rules, community assembly, cooccurrences, coral reef fish, guild proportionality

## Abstract

Coral reef fish communities are species‐rich with seemingly high apparent niche overlap. Although competition among fish species is well documented at local scales and within specific species, the extent to which it affects community structure and composition remains unclear. Here, we examine whether competition‐based models of community assembly can explain patterns of species occurrences within feeding guilds in a lagoonal reef fish metacommunity at One Tree Island on the southern Great Barrier Reef, and thus infer whether competitive exclusion is operating within guilds. Using presence–absence data from underwater fish surveys, we partitioned the fish community into feeding guilds and applied null‐model analyses to test for evidence of competition using two classical assembly rules: Diamond's co‐occurrence rule and Wilson's guild proportionality rule. Neither assembly‐rule prediction was supported. Species within guilds co‐occurred as frequently or more frequently than expected under null expectations, and guild composition varied more, rather than less, across sites. These results indicate that exclusion‐based assembly rules do not provide a strong description of guild‐level structure in this system and highlight the limits of extrapolating evidence of local competitive interactions to patterns of community assembly in species‐rich communities.

## Graphical abstract

We tested classic competition‐based community assembly predictions in a lagoonal coral reef fish community using feeding guild data and null models. Species within guilds commonly occurred together, and guild composition differed strongly among reefs. Local competition does not translate into clear community‐level structure in this system.

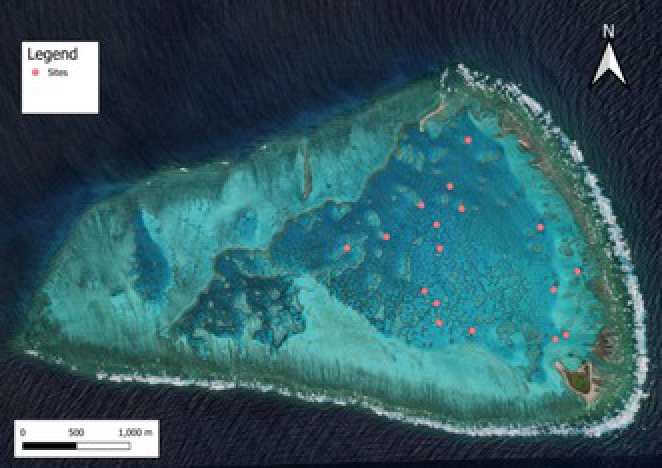

## 1 Introduction

As a key part of one of the most diverse ecosystems on the planet, coral reef fish communities comprise rich sets of coexisting species with apparent high niche overlap, historically undermining the perceived importance of competition in structuring community composition (Chesson and Warner 1981; Sale 1977). Early studies proposed that stochastic processes and environmental variability could explain species coexistence without relying on competition (Chesson and Warner 1981; Sale 1977). Sale's (1977) lottery hypothesis, for example, suggested that recruitment patterns were largely stochastic, with persistence driven by chance disturbances rather than competitive exclusion. Although empirical support for strict lottery dynamics has been limited, these ideas contributed to later views that neutral and weakly niche‐structured processes shape coral reef fish communities, as many species appear to share similar resource requirements without obvious competitive displacement (Bellwood and Hughes 2001; Ford and Roberts 2018; Munday 2004).

However, a systematic review of experimental competition studies in coral reef fishes indicates that competition may be more prevalent than previously assumed. Bonin et al. (2015) found that approximately 72% of intraspecific and 56% of interspecific tests detected significant demographic consequences, suggesting that competition may play a pervasive role in shaping species assemblages. Empirical studies have documented territoriality, interference competition and density‐dependent effects, particularly where access to limiting resources such as food, shelter or breeding sites is constrained (Forrester et al. 2006; Holbrook and Schmitt 2002; Kerry and Bellwood 2016; Robertson and Gaines 1986). Although this body of work demonstrates that competition frequently operates in reef fish systems, it primarily focuses on individual species or pairs and therefore provides limited insight into how such interactions scale to influence whole‐community structure.

While evidence of competition remains context‐specific in regulating coral reef fish communities, there is still room to question its importance in community assembly. More recent advances in community assembly theory suggest that the detectability of biotic interactions depends strongly on observational scale; biotic filtering is often most evident at finer spatial resolutions, whereas increasing environmental heterogeneity at broader scales can obscure competitive signals through stronger abiotic filtering (Araújo and Rozenfeld 2014; Cadotte and Tucker 2017; de Bello et al. 2013). At the same time, analyses conducted at very fine scales risk capturing only localised interactions that may not translate into broader patterns of community assembly (Mellin et al. 2009). A key challenge in testing competition‐based models of community assembly, therefore, is to identify spatial contexts in which abiotic gradients are sufficiently constrained to allow the emergence of biotic structuring, yet remain at a scale relevant to community‐level patterns.

Coral reef lagoons represent one such context. Lagoons are relatively enclosed environments with comparatively consistent depths and hydrological conditions, within which patch reefs form discrete habitat units embedded in a matrix of sandy substrate generally unsuitable for reef fish habitation. These patch reefs provide the structural complexity required by reef fishes and create natural, spatially bounded units for examining interactions and small‐scale community structure (Komyakova et al. 2013; Olds et al. 2012). Identifying the appropriate spatial boundaries and ecological interactions that delineate communities depends strongly on resource distribution and species movement across the landscape (Mellin et al. 2009). As such, lagoonal reef systems offer an opportunity to examine whether biotic filtering among ecologically similar species inhabiting these patch reefs leaves detectable patterns at the metacommunity scale under relatively homogeneous abiotic conditions.

While coral reef fish assemblages have been examined within spatially structured reef systems using functional‐group frameworks (Dubuc et al. 2023; Pinheiro et al. 2023), explicit tests of competitive exclusion–based assembly rules within feeding guilds remain scarce. Recent syntheses emphasise that, despite widespread evidence for biotic interactions in reef fishes, their translation into predictable community‐level assembly patterns is rarely explicitly evaluated (Bonin et al. 2015; Hodge and Price 2022). It remains unclear whether feeding guild composition in coral reef fish metacommunities conforms to predictions derived from the competitive exclusion principle when embedded within a spatially connected landscape.

In this study, we tested whether competition limits fish composition within a lagoonal coral reef fish metacommunity by examining whether assemblage patterns conform to those predicted by classical community assembly rules models derived from the competitive exclusion principle. Specifically, we evaluated Diamond's cooccurrence rule and Wilson's guild proportionality rule. Diamond's rule predicts that competitively interacting species pairs should co‐occur less frequently than expected by chance (Diamond 1975), whereas the guild proportionality rule predicts that competition within guilds constrains variation in guild composition across communities (Wilson 1989). To test these predictions, we surveyed fish communities, collected presence–absence data, partitioned the assemblage into feeding guilds to delineate putative direct competitors (Simberloff and Dayan 1991; Wilson 1989), and applied null‐model analyses based on these assembly rules. As these assembly rules are defined in terms of species occurrence and community membership, rather than abundance variation that may reflect demographic, behavioural or sampling processes independent of exclusionary dynamics, they are appropriately evaluated using presence–absence information. Accordingly, we tested the hypotheses that (1) within‐guild species cooccurrence would be lower than expected under null models, indicative of competitive exclusion and (2) variance in guild composition across communities would be reduced relative to null expectations, indicating regulation of guild membership by competition. By directly evaluating exclusion‐based assembly predictions, this study explores how competition rules at local scales translate into, or fail to translate into, patterns of community structure in species‐rich reef ecosystems.

## 2 Methods

### 2.1 Study Location and Sampling Design

**FIGURE 1:**
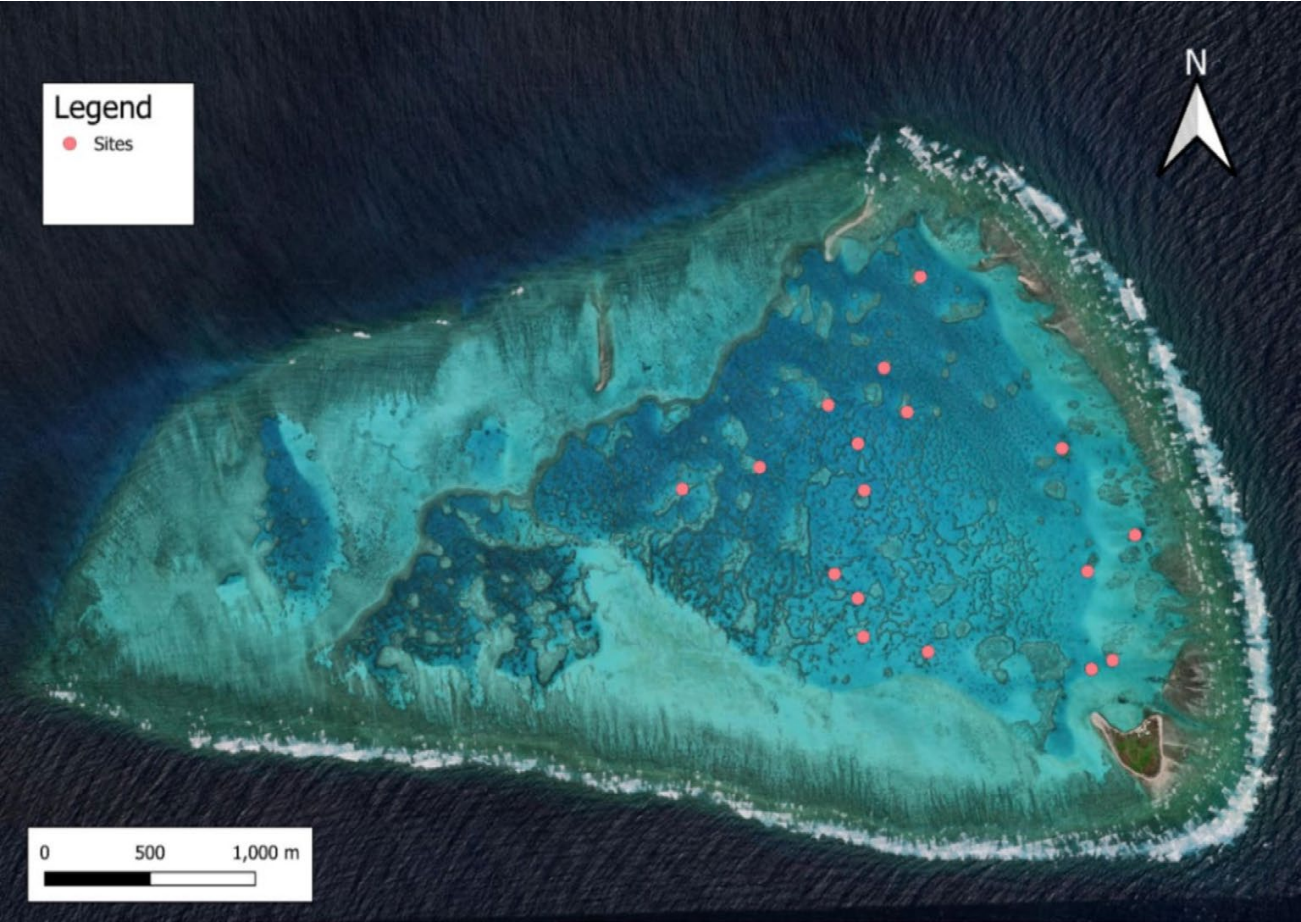
Satellite image of One Tree Island located in the southern Great Barrier Reef, Australia (23°30′ S, 152°06′ E) showing the surveyed patch reef sites (*n* = 17) within First Lagoon.

This study was conducted within the lagoonal reef system of One Tree Island during 2023. One Tree Island is a coral cay on a platform reef in the Capricorn–Bunker region of the southern Great Barrier Reef, Australia (23°30′ S, 152°06′ E) (Figure 1). The interior reef comprises a large central lagoon (First Lagoon) and two smaller lagoons (Second and Third Lagoons), partitioned by reef flats and sand sheets (Ludington 1979). The lagoons contain numerous patch reefs (micro‐atolls) composed of aggregated corals and coralline algae, with steep outer walls that descend to depths of up to 8 m (Chazottes et al. 2017; Frith and Mason 1986). A continuous reef crest surrounds the island and emerges approximately 0.4 m above sea level at low tide, isolating the lagoonal system from surrounding waters for 5–6 h during each tidal cycle (Chazottes et al. 2017; Hatcher and Frith 1985).

First Lagoon was treated as the experimental unit, with patch reefs within serving as discrete sample units. Patch reefs (*n* = 17) were selected within First Lagoon using satellite imagery and ground‐truthing to verify reef boundaries and isolation from nearby reefs. Selection criteria required reefs to cover < 1 hectare, be spatially distributed within the lagoon and be separated from nearby reefs by sand to minimise confounding effects from reef‐scale variation, and to provide ecologically discrete sampling units as much as possible.

### 2.2 Fish Community Surveys

Fish communities were surveyed by two SCUBA divers using underwater visual census methods following Method 1 of the Reef Life Survey protocol, a globally standardised, non‐destructive method for comparing observable reef‐fish assemblages across sites (Edgar and Stuart‐Smith 2014; RLS 2024). At each patch reef, a single 50 m transect was established along the exterior perimeter wall approximately midway between the reef crest and reef base. Transect placement was randomised by selecting a random start bearing between 0° and 360°, from which transects were laid clockwise along the reef perimeter. All visible and identifiable fish species observed within 5 m on either side of the transect line were recorded, covering a total area of 500 m^2^ per reef. Cryptobenthic species were retained when detected, but we do not interpret the survey as a targeted or complete inventory of cryptobenthic diversity. Each patch reef was surveyed once.

### 2.3 Data Analyses

We carried out all data manipulations and analyses in R version 4.5.0 (R Core Team 2025).

#### 2.3.1 Guild Identification and Species Guild Delineation

We assigned species to feeding guilds following the trophic classifications of Parravicini et al. (2020). In that study, trophic guilds were identified using bipartite network analysis of gut content data for 615 coral reef fish species, with modules detected by maximising network modularity. The resulting guilds included sessile invertivores, herbivores–detritivores–microvores (HMDs), corallivores, piscivores, microinvertivores, macroinvertivores, crustacivores and planktivores (Parravicini et al. 2020). Bayesian phylogenetic modelling incorporating species phylogeny and maximum body size was used to infer guild membership for species lacking gut content data (Parravicini et al. 2020).

**FIGURE 2:**
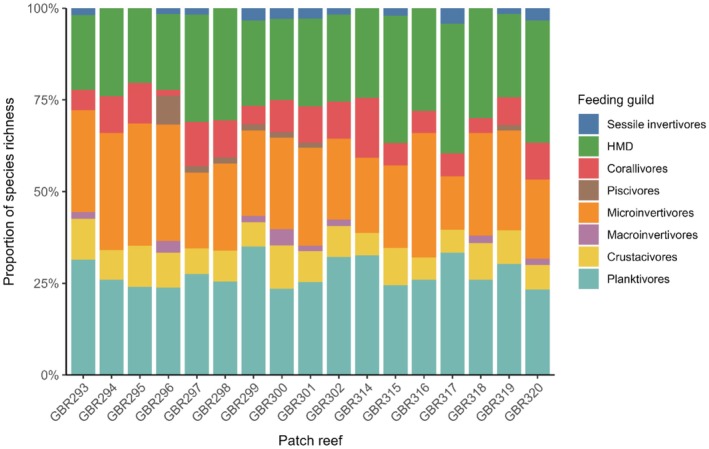
Feeding‐guild composition across surveyed patch reefs in the One Tree Island lagoonal reef system. Bars show the proportion of species richness at each patch reef assigned to each feeding guild, based on presence–absence records.

Guilds containing few species with low site occupancy (Sessile invertivores, Piscivores and Macroinvertivores) were excluded from analyses due to insufficient statistical power (Gotelli 2000; Gotelli and Ulrich 2012) (Table 1; Figure 2). Additionally, piscivore species observed during field surveys were generally highly mobile, with encounters appearing transient. Some larger predatory species are likely to move over spatial scales larger than individual transects, which may complicate their interpretation as patch‐reef residents, further justifying their removal from analysis. Furthermore, we retained the HMD guild for completeness, as it represents an independently derived trophic grouping in the Parravicini et al. framework; however, this group may include species with distinct feeding modes and/or species whose gut contents reflect mixed diets such as grazers of the epilithic algal matrix, and therefore may not correspond to a single fine‐scale competitive guild (Wilson et al. 2003). The HMD category should be interpreted cautiously.

**TABLE 1:** The number of species within each guild and the total number of sites each guild is found in across the One Tree Island lagoonal metacommunity.

Guild	Species richness	Sites present in out of 17
Sessile invertivores	3	11
Herbivores, microvores and detritivores (HMD)	46	17
Corallivores	12	17
Piscivores	7	7
Microinvertivores	40	17
Macroinvertivores	6	8
Crustacivores	18	17
Planktivores	35	17

#### 2.3.2 Null Model Analysis: Cooccurrences

We evaluated species co‐occurrence using a null‐model framework, based on Diamond's assembly rules (Diamond 1975; Gotelli and McCabe 2002). Presence–absence matrices were constructed separately for each guild, with rows representing species and columns representing patch reefs. Cooccurrence was quantified using the checkerboard score (*C*‐score), which measures the extent to which species pairs occur together (Stone and Roberts 1990). *C*‐scores were calculated for each matrix by first calculating the checkerboard unit for each species pair as:

$$C\mathrm{ᵢⱼ}=\left(\mathrm{Rᵢ}-S\mathrm{ᵢⱼ}\right)\;\left(\mathrm{Rⱼ}-S\mathrm{ᵢⱼ}\right)$$

where *R*_i_ and *R*_j_ are the number of sites occupied by species *i* and species *j*, respectively, and *S*_i,j_ is the number of sites shared by both species. The *C*‐score for each guild was calculated as the average *C*_i,j_ across all species pairs (Gotelli and McCabe 2002; Stone and Roberts 1990). For each guild, null communities of 10,000 random matrices were generated using the SIM9 fixed–fixed randomisation algorithm, which preserves both species occurrence frequencies and site richness, as implemented in the EcoSimR package (Gotelli et al. 2015; Gotelli 2000; Gotelli and Ulrich 2012). Statistical significance was assessed using two‐tailed permutation tests based on the proportion of null values more extreme than the observed statistic. To account for the influence of matrix geometry and enable standardised interpretation, results were expressed as standardised effect sizes (SES) (Gotelli 2000; Gotelli and Ulrich 2012), calculated as:

$$\begin{array}{c} \mathrm{SES}=\left(\text{observed}\;C-\text{score}-\text{Expected}\;C-\text{score}\right)/\\ \text{standard deviation of the null}\;C-\text{score distribution}. \end{array}$$

#### 2.3.3 Null Model Analysis: Guild Proportionality

We quantified guild proportionality by comparing observed variance in guild‐proportional richness among patch reefs with null‐model expectations, following Wilson's guild proportionality rule (Wilson 1989). For each patch reef, guild proportional richness was calculated as the ratio of guild species richness to total species richness, and observed variance in guild proportional richness across reefs was calculated for each guild. Expected variances were generated using a null model consisting of 1000 simulated community matrices. Null communities were generated using a fixed‐marginal randomisation implemented via swap‐based algorithms in the Vegan package (Oksanen et al. 2025). This randomisation preserves species occurrence frequencies and site richness while allowing species composition to vary freely, consistent with the assumptions of the guild proportionality rule (Wilson 1989). Expected variances were averaged across simulations to produce the null expectation. Observed and expected variances were compared using the relative variance of guild proportions index (RVgp), calculated as:

RVgp=Observed variance/Mean null variance.

Values of RVgp < 1 indicate reduced variance consistent with regulation by competitive exclusion, whereas values of RVgp > 1 indicate excess variability potentially driven by site heterogeneity or other influences (Wilson et al. 1987). Statistical significance was assessed using two‐tailed permutation tests based on the proportion of null values more extreme than the observed statistic.

## 3 Results

**FIGURE 3:**
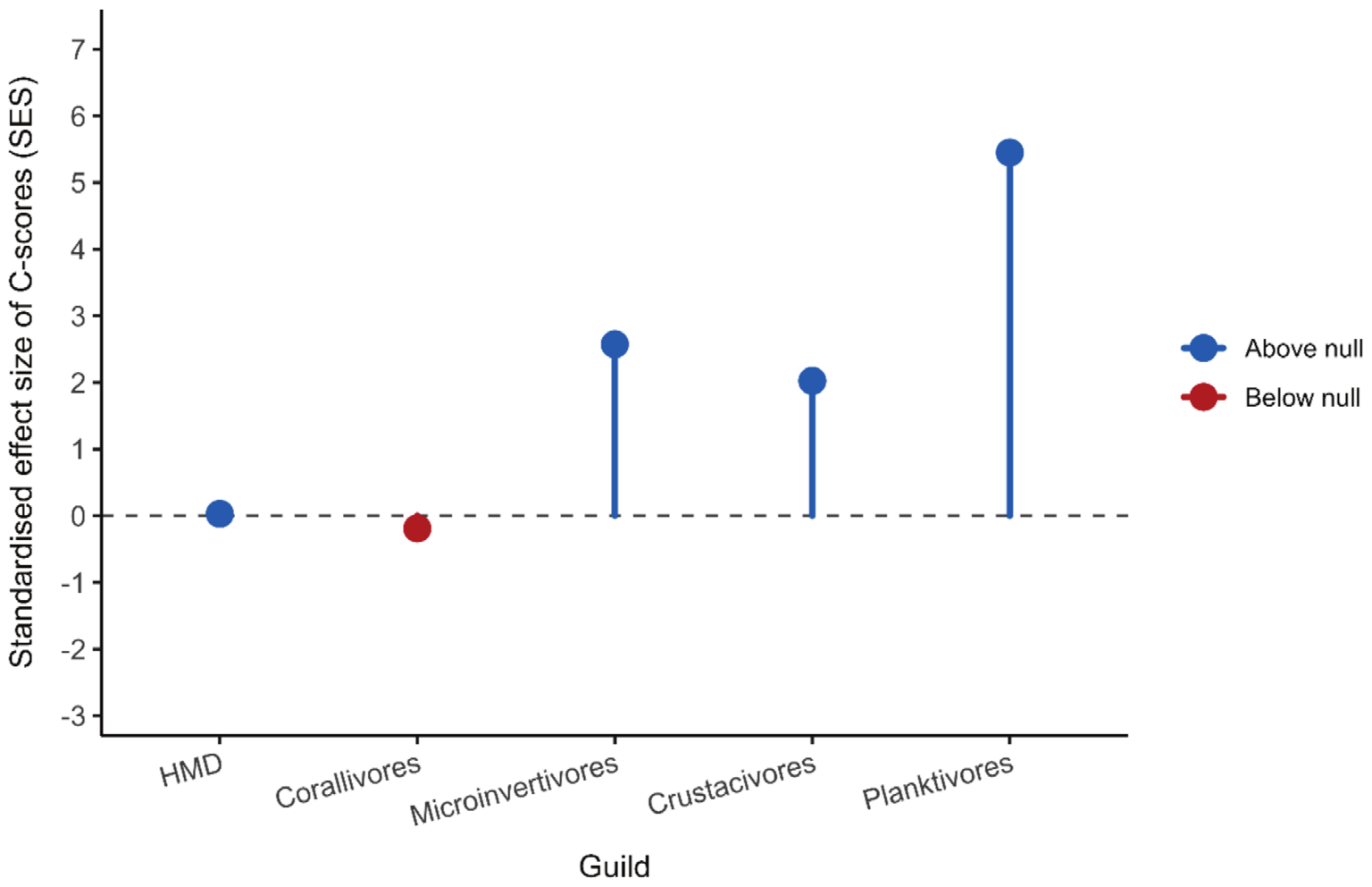
Values show the standardised effect sizes (SES) of *C*‐scores for species cooccurrence within feeding guilds across patch reefs in the One Tree Island lagoonal reef system expressed relative to the null distribution. Positive SES values indicate greater‐than‐expected cooccurrence, whereas negative values indicate segregation relative to null expectations. The dashed horizontal line denotes the null expectation (SES = 0).

No guild showed patterns consistent with competitive exclusion under either assembly rule. SES of the *C*‐score were positive for most guilds, indicating greater‐than‐expected co‐occurrence relative to null expectations (Figure 3). Planktivores exhibited the largest positive deviation (SES = 5.45, *p* = 0.0001), followed by microinvertivores (SES = 2.57, *p* = 0.013) and crustacivores (SES = 2.02, *p* = 0.045). Herbivores, microvores and detritivores (HMD) showed values close to null expectations (SES = 0.03, *p* = 0.983), while corallivores were slightly negative but also close to null expectations (SES = −0.19, *p* = 0.851). No guild exhibited significant negative SES values indicative of segregation. Hypothesis 1 was therefore not supported.

**FIGURE 4:**
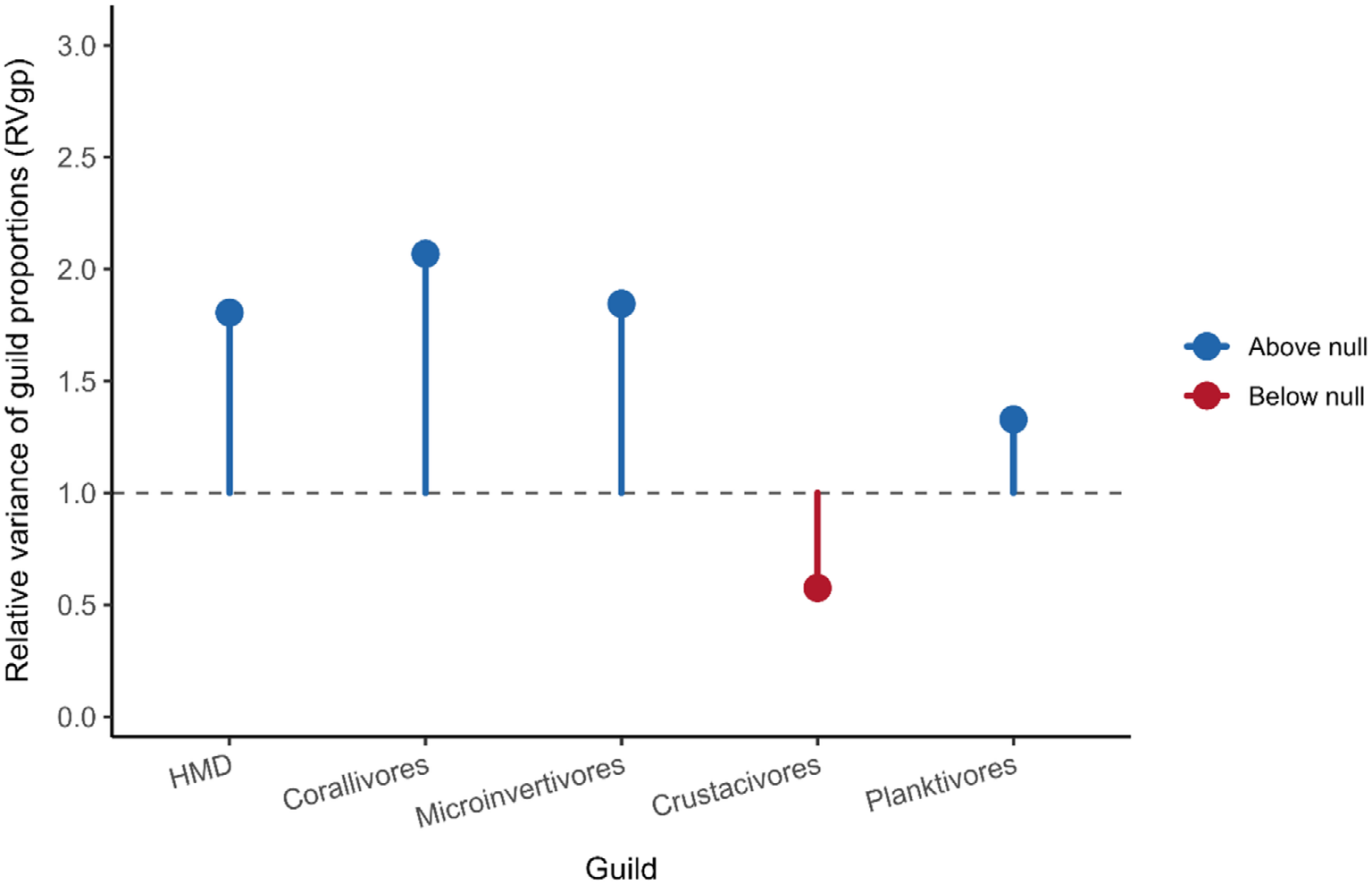
Relative variance of guild proportions (RVgp) across patch reefs for each feeding guild in the One Tree Island lagoonal reef system. The RVgp is the observed variance in guild proportional richness divided by the mean variance expected under the null model. Values of RVgp = 1 indicate agreement with null expectations, values < 1 indicate reduced variance consistent with competitive regulation, and values > 1 indicate excess variability among sites. The dashed horizontal line denotes the null expectation (RVgp = 1).

Relative variance of guild proportions (RVgp) exceeded null expectations for several guilds, indicating greater‐than‐expected variation in guild composition across sites (Figure 4). Significant excess variance was detected for HMD (RVgp = 1.81, *p* = 0.025), corallivores (RVgp = 2.07, *p* = 0.008) and microinvertivores (RVgp = 1.85, *p* = 0.017). Planktivores did not differ from null expectations (RVgp = 1.33, *p* = 0.354). Crustacivores showed RVgp < 1 (RVgp = 0.58) but this did not differ significantly from null expectations (*p* = 0.195). Hypothesis 2 was therefore not supported.

## 4 Discussion

Overall, we found that assemblage patterns in this lagoonal coral reef fish metacommunity are not structured by dietary competition as predicted by the assembly‐rules models we tested. Within guilds, species co‐occurred more frequently across sites than expected under null models, refuting our first hypothesis and contradicting predictions from classical niche theory and Diamond's assembly rules that competitive exclusion should inhibit such patterns (Diamond 1975; Gotelli and McCabe 2002). Consistent with this, our second hypothesis, that competitive exclusion constrains guild composition, was also unsupported. Variance in guild proportions across sites generally exceeded null expectations rather than showing the reduced variance predicted under strong exclusion, suggesting that site‐specific heterogeneity or other factors may influence how guild composition varies among sites (Gravel et al. 2011; Wilson 1989). The suggested site‐specific heterogeneity should be interpreted in the context of the patch‐reef design. Although patch reefs were sampled as discrete assemblages, reefs within the lagoon may still differ in position, hydrodynamic exposure, larval supply or benthic structure; such unmeasured differences could contribute to spatial autocorrelation in species co‐occurrence or guild proportions (Frith and Mason 1986; Komyakova et al. 2013; Mellin et al. 2009). Thus, while the patch‐reef design provides an appropriate scale for testing whether exclusion‐based assembly‐rule patterns are detectable, the analyses cannot fully separate those patterns from spatially structured environmental variation. These results do not preclude competitive interactions within the metacommunity but indicate that their aggregate signature does not significantly structure guild‐level patterns across the metacommunity examined here.

An additional pattern in the cooccurrence results is that feeding guilds exhibited substantial variation in effect size, aligning with differences in how guilds are expected to be coupled to patch reefs. Planktivores showed the strongest positive co‐occurrence signal, consistent with a guild whose primary resources are not confined to benthic territories or individual reef patches, but are instead structured by water‐column processes operating at broader lagoonal scales (Gove et al. 2016). Microinvertivores and crustacivores showed intermediate effect sizes, suggesting that guilds using benthic but mobile or patchily distributed prey may show partial coupling to patch‐reef habitat, potentially reflecting shared responses to habitat structure or prey availability rather than direct exclusion (Komyakova et al. 2013; McCormick 1995). In contrast, corallivores and HMDs showed weaker departures from null expectations, although likely for different reasons: corallivores may be more directly constrained by the availability and taxonomic composition of coral resources (Cole et al. 2008), whereas the HMD guild may combine species with different feeding modes and degrees of resource overlap, reducing the expectation that it should behave as a single fine‐scale competitive guild (Parravicini et al. 2020). These guild‐level differences provide an exploratory basis for future work testing whether assembly‐rule signals vary with how closely feeding behaviour and resource use correspond to the spatial scale of analysis (Araújo and Rozenfeld 2014; Götzenberger et al. 2012). Variance in guild composition followed different patterns, suggesting that differences in habitat coupling may influence species overlap and among‐site turnover in distinct ways.

Whether reduced spatial coupling dampens exclusionary dynamics or whether weaker exclusion permits greater overlap where resources are diffuse cannot be resolved from these patterns alone. Cooccurrence and variance metrics can reflect multiple interacting processes, and their interpretation as evidence for or against specific mechanisms should therefore be made cautiously (Blanchet et al. 2020). Future work could build from this spatial baseline by testing whether the lack of exclusion‐based structure persists across seasons, years, recruitment variation, disturbance or habitat change (Shulman 1985). Targeted dietary, stable‐isotope, behavioural or experimental approaches would also help determine whether positive co‐occurrence reflects shared environmental responses, realised trophic overlap, facilitation or competitive effects that do not result in local exclusion (Newsome et al. 2007; Skinner et al. 2022).

As our analyses were explicitly designed to test assembly‐rule predictions derived from the competitive exclusion principle, related theoretical frameworks are considered here only insofar as they help interpret the absence of exclusionary patterns. In this context, the lack of strong competitive exclusion signals can, in principle, be reconciled with mechanisms proposed by both modern niche theory and coexistence theory (Letten et al. 2017). Modern niche theory can explain coexistence when species differ across several niche dimensions or perform best under different local conditions (Chase and Leibold 2009; Gravel et al. 2011). In these scenarios, competing species may overlap spatially without one consistently excluding the other. Similarly, coexistence theory identifies stabilising mechanisms—including the storage effect, relative nonlinearity of competition and frequency‐dependent predation—that can reduce competitive asymmetries and allow competing species to persist under fluctuating environmental conditions (Chesson 2000). These mechanisms have empirical support, including observations in coral reef fish, demonstrating that competitive interactions do not necessarily translate into local‐scale exclusion (Carr et al. 2002; Hixon and Jones 2005; Pereira et al. 2015; Shulman 1985). Resolving such dynamics lies beyond the scope of the occurrence‐based assembly‐rule tests used here. Although presence–absence data are appropriate for testing whether competitive exclusion produces patterns of co‐occurrence or guild membership across sites, they cannot determine whether competition affects relative abundance, dominance, behaviour, growth or demographic performance without causing local absence. Addressing those questions would require abundance‐based or demographic modelling frameworks and is therefore beyond the scope of the present study.

However, the extent to which such mechanisms scale beyond local or pairwise interactions to structure entire metacommunities remains unclear, particularly in species‐rich systems where interactions are numerous, likely indirect, and strongly context‐dependent (Barabás and D'Andrea 2020; Barabás et al. 2018; Spaak and Schreiber 2023). This reflects a limitation of the modelling assumptions used to formalise these theories: interactions are often treated as stable and separable, even though their effects may be modified by feedback that emerges only within multispecies communities. As a result, predictions at the community level often remain ambiguous, making it difficult to distinguish between reduced competitive exclusion and broader forms of coexistence driven by indirect or higher‐order effects. This ambiguity is increasingly recognised as a general limitation of pattern‐based inference in complex ecological communities (Blanchet et al. 2020).

An important direction for future research and theoretical development is to examine whether alternative frameworks can better accommodate the potential indirect effects, context dependence and multispecies dynamics suggested by the observed patterns. Network‐based approaches grounded in a complexity‐theory paradigm offer one such emerging avenue by treating ecological communities as irreducible systems characterised by strong interdependence among species rather than as collections of independent pairwise relationships (Bascompte 2007). Within this framework, alternative models of species coexistence, such as intransitive competition and higher‐order interactions, are not viewed as extensions of pairwise dynamics, but as intrinsic properties of interaction networks that can stabilise high diversity through non‐hierarchical structure and feedback (Levine et al. 2017; Vandermeer 2024). Empirical work in terrestrial and marine systems, including coral reefs, provides examples of such non‐hierarchical interactions, particularly where competitive outcomes form loops or where the effect of one species depends on the presence of others (Precoda et al. 2017; Sinervo and Lively 1996; Soliveres and Allan 2018). At present, however, these approaches should be viewed as complementary and hypothesis‐generating rather than explanations for the patterns observed here. Developing and applying methods capable of detecting network‐level dynamics at the community and metacommunity scales, therefore, represents an alternative path for future studies seeking to understand coexistence in species‐rich systems where exclusion‐based assembly rules may not apply.

## 5 Conclusion

Despite the patch reef of the lagoon representing habitats with the highest potential for interspecific competition, our analyses did not support the specific competitive‐exclusion‐based assembly‐rule predictions tested in this lagoonal patch‐reef fish metacommunity. Species within feeding guilds were not spatially segregated across patch reefs; instead, several guilds co‐occurred more frequently than expected, and guild proportional richness often varied more among reefs than predicted under null expectations. These results do not rule out competition, but indicate that any competitive effects did not produce the occurrence‐based signatures expected under Diamond's co‐occurrence rule or Wilson's guild proportionality rule at the spatial and guild‐level scale examined. This finding may have broader relevance for other species‐rich reef systems where high diversity, variable resource distributions or scale‐dependent habitat structure complicate simple expectations of spatial exclusion. Testing this possibility will require targeted comparisons across contrasting reef habitats, regions and environmental gradients, including lagoonal reefs, outer reef slopes and deeper reef habitats. Overall, our findings caution against assuming that local competitive interactions necessarily translate into detectable exclusion‐based assembly patterns at the community or metacommunity level.

## Author Contributions

**Simon P. Um:** conceptualization (equal), formal analysis (lead), investigation (equal), methodology (equal), writing – original draft (lead), writing – review and editing (equal). **John W. Turnbull:** conceptualization (equal), formal analysis (supporting), investigation (equal), methodology (equal), writing – review and editing (equal). **Emma L. Johnston:** conceptualization (equal), funding acquisition (equal), writing – review and editing (supporting). **Graeme F. Clark:** conceptualization (equal), formal analysis (supporting), funding acquisition (equal), investigation (equal), methodology (equal), writing – review and editing (equal).

## Funding

This work was supported by Australian Research Council grant, SR200100005 Securing Antarctica's Environmental Future.

## Conflicts of Interest

The authors declare no conflicts of interest.

## Supporting information


**Data S1:** ece374011‐sup‐0001‐DataS1.zip.

## Data Availability

Original data that support the findings of this study are available in the Supporting Information of this article. Data reuse: Additional data used in this study includes a previously published dataset from Parravicini et al. (2020).
